# Pool of items to measure Primary Health Care workers’ knowledge on healthy eating

**DOI:** 10.11606/s1518-8787.2021055003218

**Published:** 2021-11-08

**Authors:** Lígia Cardoso dos Reis, Patricia Constante Jaime

**Affiliations:** I Universidade de São Paulo Faculdade de Saúde Pública Programa de Pós-Graduação em Nutrição em Saúde Pública São PauloSP Brasil Universidade de São Paulo. Faculdade de Saúde Pública. Programa de Pós-Graduação em Nutrição em Saúde Pública. São Paulo, SP, Brasil; II Universidade de São Paulo Faculdade de Saúde Pública Núcleo de Pesquisas Epidemiológicas em Nutrição e Saúde São PauloSP Brasil Universidade de São Paulo. Faculdade de Saúde Pública. Núcleo de Pesquisas Epidemiológicas em Nutrição e Saúde. São Paulo, SP, Brasil

**Keywords:** Health Personnel, Health Knowledge, Attitudes, Practice, Diet, Healthy, Surveys and Questionnaires, Validation Study

## Abstract

**OBJECTIVE:**

To develop and validate a self-applicable instrument for measuring primary health care (PHC) workers’ knowledge on healthy eating.

**METHODS:**

A six-step methodological study to develop and validate a measurement instrument: item development based on the Brazilian Dietary Guidelines’ chapters; content validation with a panel of experts; face validation with potential instrument users; online instrument reevaluation by participants of the content and face validation panels; online application of the instrument with PHC workers; confirmatory factor analysis for construct validation.

**RESULTS:**

A first version with 25 items underwent content and semantic changes in the content and face validation panels, being reorganized into a second version with 22 items. In the reevaluation, participants considered 21 questions to be clear and representative of the Brazilian Dietary Guidelines, with one being excluded. This third version of the instrument underwent confirmatory factor analysis after being applied online with 209 PHC workers from all Brazilian macroregions. We excluded five items in this analysis: four due to bivariate empty cells and one due to low discrimination capacity. The final model, with 16 items loaded onto one dimension, returned good fit indices [χ^2^_(104)_ = 119.047, p = 0.1486; RMSEA = 0.026 (90% CI = 0.000 to 0.046), Cfit = 0.979; CFI = 0.924; TLI = 0.913]; its information peak was below average.

**CONCLUSIONS:**

The instrument proved to be valid and accurate for assessing PHC workers with below average knowledge of the Brazilian Dietary Guidelines. It might contribute to improving actions to promote healthy eating in Brazilian PHC settings by identifying the need for training health professionals.

## INTRODUCTION

Published by the Ministry of Health in 2014, the second edition of the Brazilian Dietary Guidelines (BDG) had the challenge of including, in its recommendations, the interface between the human right to adequate food and health promotion and disease prevention actions. Adopted in Brazil as an instrument to support programs and policies for promoting adequate and healthy eating^1^, the BDG^1^ were made available online in Portuguese, English, and Spanish, being recognized internationally for their content and for incorporating sustainability^2^. Their chapters address food choices based on a classification that includes the extent and purpose of food processing, with recommendations on culinary preparations, ways of eating, and obstacles to healthy eating, while considering social and environmental sustainability as one of their principles^3^.

Although addressed to the population as a whole, BDG target readers are health workers and other health promotion professionals, since they play a key role in disseminating messages to the population^3^. For United Nations’ Food and Agriculture Organization (FAO)^4^, dietitians are not the only health professionals who should help disseminate the guidelines; efforts should also be made for the continuing education of all health workers involved in promoting healthy eating.

Both the World Health Organization and the United Nations Children’s Fund^5^ have highlighted the potential for Primary Health Care (PHC) professionals to meet the main health needs of the population near their homes, especially regarding their demand for food. This would require qualified and (ideally) multiprofessional teams.

For Vanderlee et al.^6^, although knowledge is only one of many factors influencing food intake, it would be unrealistic to expect people to follow the recommendations of dietary guidelines without knowing their content. Although FAO^4^ has recognized dietary guidelines as the expression of food and nutrition education principles, to date, no tools are available in the literature to assess health workers’ knowledge on their content. Since the publication of the BDG, only one instrument has been published recently to assess the population’s adherence to their recommendations^7^.

Thus, this study aims to develop and validate an instrument to measure PHC professionals’ knowledge about the content of the BDG.

## METHODS

### Theoretical Background

This paper describes the process of developing and validating a self-applied online instrument to measure PHC professionals’ knowledge about the BDG content.

The literature highlight knowledge of food and nutrition as an attribute of food literacy^8,9^. According to the Social Cognitive Theory^10^, developed by Albert Bandura, this concept is a precondition for a shift towards health promotion through social cognitive means, thus helping individuals to find reasons for behavior change. A systematic review by Krause et al.^9^ points out that the central conceptual framework of food literacy includes practical knowledge and skills to regulate food intake, such as meal planning, food selection and preparation.

The BDG^1^, theoretical framework used to develop the items of the instrument, recommends choosing and planning meals autonomously and critically, while recognizing the social, cultural, environmental, and economic dimensions of healthy eating.

Its first chapter (*Principles*) presents the guiding principles of their recommendations. Chapter 2 (*Choosing foods*) makes general recommendations for choosing fresh or minimally processed foods as the basis of diets, showing a classification of foods based on the extent and purpose of processing. Chapter 3 (*From foods to meals*) provides advice on how to combine foods in meals, based primarily on fresh and minimally processed foods. Chapter 4 (Ways of eating) addresses the circumstances that influence food metabolism and the pleasure of eating. Chapter 5 (*Understanding and overcoming obstacles*) lists potential obstacles to proper and healthy eating. A summary of the main BDG recommendations can be found at the end of the publication, listed as “*Ten Steps to Healthy Diets”*^1^.

### Instrument Development and Validation

We adopted a six-step methodological procedure to develop and validate the instrument: item development (step 1); content validation with a panel of experts (step 2); face validation by a focus group of potential instrument users (step 3); online instrument reevaluation by the participants in steps 2 and 3 (step 4); online application of the instrument to PHC professionals (step 5); confirmatory factor analysis for construct validation (step 6).

We first developed a 25-item instrument (step 1), each with a three response option (*True*; *False; Do not know*), to cover the five chapters of the BDG^1^.

Eight experts were then invited to analyze the instrument and validate its content (step 2) in a face-to-face panel, but only seven attended: four BDG experts, one psychometrics expert, one health promotion expert, and one food and nutrition education expert. All participants were familiar with the BDG and received, in addition to a copy of this document, the first version of the instrument.

The experts were instructed to relate each item on the instrument to at least one chapter of the BDG, rating each item based on representativeness (1 = item is not representative of the BDG; 2 = item needs major revisions; 3 = item needs minor revisions; 4 = item is representative of the BDG content) and clarity (1 = item is unclear; 2 = item needs major revisions; 3 = item needs minor revisions, 4 = item is clear). Following Hall et al.’s method^11^, we also asked the experts to explain their reasons for not considering a particular item representative or clear, as well as to suggest changes and provide additional comments on repetition, difficulty and adequacy of the instrument to measure the proposed construct.

We calculated an average rating for representativeness and clarity, removing items with score lower than 3.0 from the instrument; items scoring between 3.0 and 4.0 were either removed or edited based on the experts’ comments and suggestions after concluding step 3 (face validation).

This third step aimed to measure whether the items are appropriate “*at face value,*” that is, if they are clear enough to their target audience^12^. Eight potential instrument users (health professionals with previous PHC experience) were invited to validate the first version, but only six participated: three dietitians, one physical educator, one doctor and one speech therapist. The instrument was qualitatively evaluated by this focus group regarding its structure, response pattern, method of application, usefulness, and any semantic changes to make the items clearer. Participants for steps 2 and 3 were selected according to Nunally & Bernstein^13^to obtain a sample of professionals from different fields related to the purpose of the instrument (content validation) and primary health care professionals that could assess its clarity (face validation).

After the assessment and considerations made by the content and face validation panels, the instrument underwent changes resulting in a second version. This corrected version was sent electronically, via Google Forms, to the same experts and health professionals (step 4).

The participants were instructed to evaluate the instrument using the same method as the content validation step, assigning a score from 1 to 4 for the representativeness and clarity of the items. The platform also offered fields for comments on the items and the instrument as a whole.

We received six assessments from the participants: three from the content validation panel and three from the face validation panel. The average scores assigned to each item were calculated using the same method for excluding/revising the items from the content validation step.

After making adjustments based on the suggestions received, we applied a third and final version of the instrument to a sample of PHC professionals with at least 1 year of experience in this field (step 5) calculated by estimating five to ten observations per variable (item), as recommended by Nunally & Bernstein^13^.

Respondents were recruited using the researchers’ social media and the university’s website until we reached the desired sample size. A website was designed and programmed to record the IP address of respondents’ computers, thus avoiding duplicate entries, besides providing information on the research and fields for the respondent to fill with personal and professional data. The website was launched in April 2017, and data collection for construct validation of the instrument with confirmatory factor analysis (step 6) was completed in June 2017.

### Data Analysis

Confirmatory factor analysis was used to provide construct validity of the instrument, which consisted of dichotomous items (correct/incorrect responses); thus, answers recorded as “*I don’t know*” were coded as incorrect. The analysis assumed that the items, as a whole, would respond to a single dimension (knowledge of the BDG).

Categorical data was analyzed by an appropriate estimator^14^, where the weighted least squares estimator (WLSMV) estimates the magnitude of factor loadings more accurately when compared with maximum likelihood estimators^15^.

The models were run on Mplus version 8.0, with goodness of fit being assessed by the CFI (Comparative Fit Index), TLI (Tucker-Lewis Index) and RMSEA (Root Mean Square Error Approximation) indices. The goodness of fit cut-off points were CFI and TLI > 0.90, and RMSEA < 0.08^16^. RMSEA close fit (Cfit) values above 0.05 indicated a good fitted model.

Item Response Theory (theta parameterization) was used to estimate the discrimination (*a* parameter ) and difficulty (*b* parameter) item parameters, with cut-off points available in Baker & Kim^17^. Since the respondents lived in different macro-regions of Brazil (non-independent/multilevel structure), we considered the proposal of Asparouhov^18^ and Asparouhov^19^ for the standard errors and the Chi-square test of the model fit.

The total information curve was designed to identify at which interval of the evaluated construct (knowledge) the instrument works best.

This research was approved by the Research Ethics Committee of the School of Public Health, University of São Paulo, under protocol number 56303716.6.0000.5421. Participation was voluntary and all participants signed an informed consent form.

## RESULTS

### Instrument Development and Content and Face Validation

Our first instrument comprised 25 items. After the content and face validation panels, we excluded fours items, reworded 18 based on the comments made by the experts, and added one item to include content from chapter 4 of the BDG (Ways of eating). The changes resulted in a second version of the instrument with 22 items.

This second version underwent re-evaluation by the participants of the content and face validation panels. All experts found the instrument items clear and representative of the BDG content, excepting item 16 (*Beans prepared with instant seasoning - powders or broths in tablet form - have more sodium than beans made with salt, garlic and bay leaf.*), which was excluded for its averages below 3.0 for clarity and representativeness. Its third version thus comprised 21 items (see additional file) and underwent construct validation with factor analysis.

### Construct Validation

We invited health professionals who have been providing PHC services in Brazil for at least 1 year to answer the instrument on the research website, collecting data from 209 PHC workers from all macroregions of Brazil (Table 1).

**Table 1:** Characteristics of participants in the construct validation of the instrument. São Paulo, 2017.

Variables	Total no. of respondents (n = 209)

	n (%)
Gender	
Female	191 (91.4)
Male	18 (8.6)
Age (years)	
≤ 25	6 (2.9)
26–35	80 (38.3)
36–45	78 (37.3)
46–54	37 (17.7)
≤ 55	7 (3.3)
Did not answer	1 (0.5)
Profession	
Dietitian	133 (63.6)
Other health workers^a^	76 (36.4)
Schooling level	
Graduate	165 (78.9)
Undergraduate	34 (16.3)
No degree	10 (4.8)
Length of experience in Primary Health Care	
< 5	72 (34.4)
5–10	72 (34.4)
> 10	65 (31.1)
Workplace - Regions of Brazil	
North	8 (3.8)
Northeast	36 (17.2)
Midwest	14 (6.7)
Southeast	109 (52.2)
South	42 (20.1)

^a^ Other health professionals = 32 nurses, 8 doctors, 8 community health agents, 5 social workers, 5 speech therapists, 5 psychologists, 5 physical educators and 8 other health professionals.

The initial 21-item model loaded onto one dimension returned empty bivariate cells between some pairs of items. Empty bivariate cells imply a perfect correlation (r = 1) and therefore one of the items – in which this perfect correlation is observed – should be excluded. We thus excluded items 1, 7, 19 and 20 (see additional file) to make the model admissible.

After an initial inspection, the model returned excellent fit indices – χ^2^_(119)_ = 137.774, p = 0.114; RMSEA = 0.027 (90% CI = 0.000 to 0.046), Cfit = 0.982, CFI = 0.905, TLI = 0.891 –, although TLI was below the cutoff point (> 0.90) for a model with good fit. After inspecting the discrimination parameters for the remaining 17 items, we found that item 12 “*Intake of rice and beans for lunch or dinner increases satiety*” had the lowest discriminant value according to the degree of knowledge (a = 0.521, p = 0.009). After its exclusion from the instrument, all fit indices improved and returned a well-fitting model: χ^2^_(104)_= 119.047, p = 0.1486; RMSEA = 0.026 (90% CI = 0.000 to 0.046), Cfit = 0.979, CFI = 0.924, TLI = 0.913.

Table 2 shows the discrimination and difficulty, with their respective standard errors, of the 16 items of the unidimensional model (final version of the instrument). Regarding difficulty (Table 2), 13 of the remaining 16 items of the final version of the instrument presented values below -1, indicating that they are easy to answer. Items 13 and 21 showed difficulty parameters below -3 (b = -3.630 and b = -3.5, respectively), thus being the easiest to answer, while item 2 appeared to be the most difficult (see additional file). But since its difficulty is close to zero (b = -0.211, p < 0.001), one could argue that item 2 has an average degree of difficulty.

**Table 2:** Discrimination and Difficulty of the items in the final version of the instrument. São Paulo, 2017.

Item^a^	Discrimination	Standard error	p	Difficulty	Standard error	p
Q2	0.473	0.104	< 0.001	-0.211	0.192	0.274
Q3	0.490	0.108	< 0.001	-2.673	0.502	< 0.001
Q4	1.232	0.290	< 0.001	-2.086	0.257	< 0.001
Q5	0.441	0.114	< 0.001	-1.680	0.466	< 0.001
Q6	0.422	0.082	< 0.001	-2.581	0.514	< 0.001
Q8	0.980	0.189	< 0.001	-0.969	0.168	< 0.001
Q9	1.327	0.309	< 0.001	-1.748	0.302	< 0.001
Q10	0.451	0.072	< 0.001	-0.726	0.208	< 0.001
Q11	0.984	0.136	< 0.001	-1.195	0.225	< 0.001
Q13	0.343	0.130	< 0.001	-3.630	1.204	0.003
Q14	0.782	0.121	< 0.001	-2.167	0.260	< 0.001
Q15	0.838	0.170	< 0.001	-1.331	0.275	< 0.001
Q16	0.521	0.108	< 0.001	-3.507	0.595	< 0.001
Q17	0.637	0.075	< 0.001	-1.657	0.238	< 0.001
Q18	0.655	0.226	0.004	-2.239	0.520	< 0.001
Q21	0.474	0.185	0.011	-3.500	1.245	0.005

^a^ items 1, 7, 12, 19 and 20 were excluded in the construct validation step.

The Figure shows the total information curve of the instrument, where the X-axis describes the respondents’ amount of knowledge in z-score (mean at 0) and the Y-axis shows the amount of information (accuracy) of the instrument. The instrument is more accurate (information peak) for those with below average knowledge about the BDG.

**Figure:**
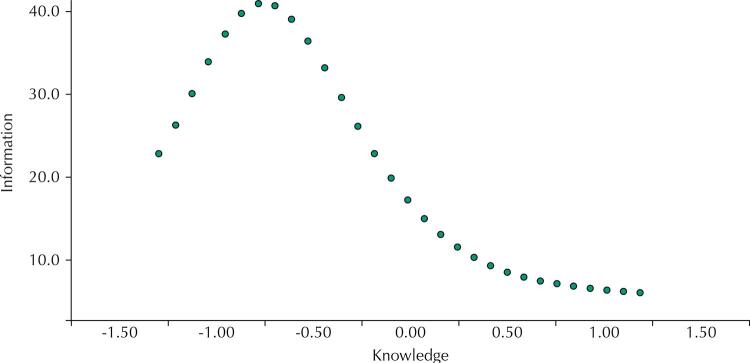
X axis describes the amount of knowledge of respondents in z-score (mean at 0) and the Y axis shows the amount of information (precision) of the instrument.

## DISCUSSION

This study developed and validated an instrument to measure PHC professionals’ knowledge of the Brazilian Dietary Guidelines^1^, which proved accurate for assessing professionals whose knowledge is below average. Such psychometric feature suggests that the tool should be used to evaluate professionals who require continuing education on promoting proper and healthy eating.

Psychometrics has been widely used as a method to develop and validate food and nutrition tools^7,20^. The content and semantic changes made before the construct validation, based on the suggestions made by experts and potential instrument users in the initial steps, resulted in an easy-to-apply 16-item instrument. After the confirmatory factor analysis, we excluded four items for model-fitting purposes: item 1 explored the BDG principles; item 7, the recommendations on meal composition; items 19 and 20 explored the obstacles “*advertising*” and “*time*” for adequate and healthy eating. Despite these exclusions, other items covering the five chapters of the guidelines remained in the instrument. The item “*Intake of rice and beans for lunch or dinner increases satiety*” had the lowest discrimination value in the pool of items, being excluded from the instrument for a good model fit. This suggests that the relationship between eating rice and beans and satiety is well known and established among respondents. As these foods are part of the traditional Brazilian dietary pattern, such an information must be empirically known to health professionals. Content on meal composition appeared in other items of the instrument.

To our knowledge, since the BDG has been published, there are no tools available in the literature to assess PHC professionals’ knowledge on healthy eating recommendations and guidelines. These tools can help PHC service managers and other parties involved in workforce training to promote proper and healthy eating when diagnosing and monitoring the implementation of dietary guidelines, as recommended by FAO^21^. For this organization, one of the lessons learned over the past decades is the need to develop mechanisms to implement dietary guidelines that go beyond visual icons, thus suggesting that policy makers develop plans for implementing, evaluating, monitoring and redesigning these tools^22^.

The qualification of health workers regarding food and nutrition education and prevention of public health problems related to food and nutrition is crucial to promote the health of the population^23^. A recent assessment of food consumption patterns from 195 nations showed that dietary improvements can prevent 1 in 5 deaths worldwide. These findings showed that poor diets may cause more deaths than any other risk factor (including smoking), indicating the urgency of population-level dietary interventions^24^.

FAO^4^ recognizes professional training as a key element to implement dietary guidelines and highlights the importance of assessing the impact of implementing these tools in Latin American and Caribbean countries. Brazil, for example, has a public policy that includes food and nutrition education in the Unified Health System through the services provided by PHC workers. Although the Brazilian food and nutrition education guidelines infer that these actions should be taken by all health workers, research shows that dietitians remain the key actors in this situation^25^.

Several studies in nations that adopt dietary guidelines in their agendas to promote adequate and healthy eating have reported poor adherence to these recommendations^26-28^. Considering the role of health professionals in disseminating information on healthy eating, as advised by dietary guidelines^4^, we expect that the instrument proposed in this study to be useful for planning and implementing continuing education activities that can change this scenario.

Brazil’s public health system stands out for the attention given in recent years to the reorganization of PHC, taking on the challenge of adopting a health care model based on health promotion^29^. The instrument proposed in this study can therefore help Brazil to reorganize activities to promote adequate and healthy food in PHC from this perspective.

Designed by the Ministry of Health as a theoretical reference for the Unified Health System to promote adequate and healthy eating, the BDG supports the development of personal skills and the reorientation of health services from the perspective of health promotion^1^. To this end, the literature suggests that health literacy should be more explicitly addressed in public policies and holistic interventions, thus ensuring that all population groups make informed and autonomous decisions^30^. As the absence of tools can hinder assessing and monitoring the health professionals’ food literacy, and evaluating interventions focused on it, the instrument proposed in this study may fill a gap in the literature regarding the availability of validated instruments to measure PHC health workers’ skills for dissemination of BDG recommendations, and may inspire researchers from other nations.

Krause et al.^9^ suggested that, in measuring food literacy, one should consider the following skills and competences: reading, understanding, and judging the quality of information; accumulating and exchanging knowledge on food and nutrition; practical skills about purchasing food and preparing meals; thinking critically about factors that influence food choices; and understanding the impact of those choices on society. Importantly, the chapters of the BDG^1^ make recommendations for critical and conscious food choice to prepare and eat healthy, tasty, culturally referenced, and socially and environmentally sustainable meals. The guidelines also present ways to overcome potential obstacles to adequate and healthy eating at the individual, collective, and public policy levels to ensure the human right to adequate food. For Vanderlee et al.^6^, the BDG stands out because its messages are less complex; people can thus more easily understand and engage with the principles of healthy eating.

An important limitation of this study was the lack of participation of nurses in the face validation step, as these professionals play an important role in PHC practices and their adherence could make a significant contribution to the construct validation step. Moreover, other parameters must be used for instrument validity. We suggest that future research focus on further analyzing the internal consistency of the instrument, since to our knowledge, this is the first study to present a validated instrument to measure PHC workers’ knowledge on the recommendations of a national food guide. The developed instrument has been shown to have content, face and construct validity to assess primary health care professionals whose BDG knowledge is below average.
